# The Biology of Autoimmune Response in the Scurfy Mice that Lack the CD4^+^Foxp3^+^ Regulatory T-Cells

**DOI:** 10.3390/biology1010018

**Published:** 2012-04-04

**Authors:** Shyr-Te Ju, Rahul Sharma, Felicia Gaskin, John T. Kung, Shu Man Fu

**Affiliations:** 1 Center for Immunity, Inflammation and Regenerative Medicine, School of Medicine, University of Virginia, Charlottesville, VA 22908, USA; 2 Department of Medicine, School of Medicine, University of Virginia, Charlottesville, VA 22908, USA; 3 Department of Microbiology, Immunology, and Cancer, School of Medicine, University of Virginia, Charlottesville, VA 22908, USA; 4 Department of Psychiatry and Neurobehavioral Sciences, School of Medicine, University of Virginia, Charlottesville, VA 22908, USA; 5 Academia Sinica, Nankang District, Taipei 11529, Taiwan; 6 Department of Immunology, School of Medicine, National Taiwan University, Taipei 10051, Taiwan

**Keywords:** Scurfy mice, regulatory T-cells, multi-organ inflammation, genetic control of MOI

## Abstract

Due to a mutation in the Foxp3 transcription factor, Scurfy mice lack regulatory T-cells that maintain self-tolerance of the immune system. They develop multi-organ inflammation (MOI) and die around four weeks old. The affected organs are skin, tail, lungs and liver. In humans, endocrine and gastrointestinal inflammation are also observed, hence the disease is termed IPEX (Immunodysregulation, Polyendocrinopathy, Enteropathy, X-linked) syndrome. The three week period of fatal MOI offers a useful autoimmune model in which the controls by genetics, T-cell subsets, cytokines, and effector mechanisms could be efficiently investigated. In this report, we will review published work, summarize our recent studies of Scurfy double mutants lacking specific autoimmune-related genes, discuss the cellular and cytokine controls by these genes on MOI, the organ-specificities of the MOI controlled by environments, and the effector mechanisms regulated by specific Th cytokines, including several newly identified control mechanisms for organ-specific autoimmune response.

## 1. Introduction

Self-tolerance is mediated by the CD4^+^Foxp3^+^ regulatory T-cells (Treg) [1,2,3,4,5]. Treg expression depends on the X-linked gene encoding the transcription factor Foxp3 [4,5]. Patients bearing a mutation in their *foxp3* gene invariably develop IPEX (immune disregulation, polyendocrinopathy, enteropathy, X-linked) syndrome, which is characterized by systemic multi-organ inflammation manifested as diarrhea, eczematous dermatitis, insulin-dependent diabetes mellitus, anemia, thrombocytopenia, neutropenia, and tubular nephropathy [6]. However, variation of symptoms is observed among families and within families [6]. The severity of the mutational effect, genetic background that influences Foxp3 expression or function, environment, and age are likely contributing factors. 

The IPEX mutations are rare and often affect different positions of the Foxp3, leading to different manifestations and severity of the autoimmune responses [6]. In contrast, the mutation in Foxp3 of the genetic homogeneous Sf mice results in total absence of Treg. Sf mice were originally derived by W.L. Russell from Oak Ridge National Lab. They were maintained in a non-inbred background [7]. Godfrey *et al*. had generated a 129/RI (H-2^b^) congenic background and a C3H/HeSnJ (H-2^b^) congenic line to study Sf inflammation [8]. Others have bred the Sf mutant gene into the BALB/c background [9]. Means *et al*. backcrossed *Foxp3^sf/+^* females to C57BL/6NTac males. The Jackson Lab received N8 mice and backcrossed to C57BL/6J to generate B6.Sf mice [10]. Because of genetic homogeneity, spontaneous autoimmune response develops in a rapid, predictable and unabated manner, leading to severe multi-organ inflammation (MOI), and death around 3 to 4 weeks of age. The major organs affected among Sf mice of various genetic backgrounds are observed in skin, lungs, liver, and stomach. Because the large repertoire of mutant gene mice is available in B6 background, B6.Sf mice are the mutant of choice to study genetic control of Sf MOI. This autoimmune inflammation provides an ideal and highly efficient model to study the autoimmune regulation controlled by Treg and various inflammation factors that regulate inflammation beyond the Treg checkpoint. 

The skin, tail, lungs and liver are affected first in Sf mice [11]. Sf mice may have the potential to develop inflammation in other organs. A low frequency of organ-specific T-cells, a limited supply of antigen (Ag), the pre-weaning condition, organ development and early death are potential reasons that affect their development. Transfer of Sf T-cells into *Rag1^−/−^* recipients induced inflammation in additional organs [11]. Severe gastrointestinal inflammation rapidly developed in neonatal *Rag1^−/−^* recipients just a few days after weaning, suggesting mother’s milk and the intestinal microbes play a role in the enteropathy [11]. Moreover, inflammation could be demonstrated in accessory reproductive organs in Sf.*Fas^lpr/lpr^* double mutant mice that lived beyond adulthood [12]. Thus, Sf mice offer a unique system to study how MOI is developed and regulated by various immune response genes and environmental changes. A frequently used approach is to breed a specific gene, usually in its mutant form, to Sf mice and then determine its effect on the autoimmune response at the organ, cellular and molecular levels. Another approach is to prolong the life span of the Sf mice by various means and study the autoimmune response under different environments.

## 2. Genetic Control of MOI in Sf Mice

### 2.1. Lymphocyte Requirement

MOI and early fatality were inhibited when *Rag1^−/−^* or *Rag2^−/−^* mutant gene was bred into Sf mice, demonstrating the critical role of lymphocytes in the fatal autoimmune responses in Sf mice [13]. 

Under normal conditions, the development of a “mature” immune system is complete by 4–6 weeks of age. In addition to genetic factors, the establishment of gut microbiota after weaning contributes to this transition. The fact that fatal MOI develops in 2–4 weeks old Sf mice indicates that a complete and competent autoimmune response system is already in place within 2 weeks of birth and that the normal maturation of peripheral immune system is constrained by Treg. 

### 2.2. T-Cell Repertoire Requirement

Because the MOI in Sf mice is mediated by polyclonal CD4^+^ T-cells, T-cell receptor (TCR) repertoire reduction by genetic manipulation impacts the disease. Breeding foreign Ag-specific TCR transgenes (Tg) into Sf mice delayed but did not eliminate the fatal MOI [13]. Importantly, TCR Tg Sf mice in *Rag^−/−^* background did not develop Sf disease because the polyclonal TCR repertoire was not generated [13]. In TCR Tg mice, Tg TCR genes greatly reduce but do not completely block endogenous TCR gene rearrangement. A substantial fraction (~30%) of their T-cells expresses dual-TCR [14,15]. The endogenous TCR repertoire derived is large enough to elicit MOI, although their quantity on a per cell basis is reduced. These considerations explain the delayed but still fatal MOI in Sf mice bearing foreign Ag-specific TCR Tg.

The power of Treg control of dual-TCR T-cell expansion was studied in the ovalbumin (OVA)_323-339_-Tg TCR in Sf mice [16]. The dual-TCR T-cells were detected at 6 days after birth and could reach to 85% of the lymphocytes. By contrast, T-cells bearing only Tg TCR were not activated because OVA_323-339_ was absent in the mice. The expansion of T-cells bearing non-Tg TCR occurred in the peripheral lymphoid tissues but not in the thymus. Importantly, transfer of T-cells that had been depleted of the Tg TCRβ into *Rag1^−/−^* mice induced MOI, suggesting MOI could be induced by T-cells bearing endogenously derived TCRβ [16]. 

Many mutant genes involved in various forms of autoimmune inflammation have been bred into Sf mice to study their impact on autoimmune manifestation. We will discuss these studies first. Recently, we have conducted additional studies on 11 mutant inflammation related genes in Sf double mutant mice [17,18]. These studies include genome-wide microarray and functional analyses. The findings and significance of the study will be discussed later.

The Th cells that are supposed to be Treg in Sf mice in fact develop into Th cells with both Th1 and Th2 subsets. They do not produce IL-2 and are dependent on conventional Th cells for survival. Other have suggested that these “Treg wannabe” cells are enriched for “autoreactivity [19,20]. 

### 2.3. *Sf*.Cd4^−/−^ and *Sf*.β2m^−/−^ Mice

Breeding *Cd4^−/−^* and *β2m^−/−^* genes into Sf mice demonstrated that *Cd4^−/−^* but not *β2m^−/−^* gene affected the mortality, organ inflammation, and immunological parameters of Sf mice, thus, implicating the critical role of CD4^+^ T-cells to the fatal disease [21]. The lifespan of Sf.*Cd4^−/−^* mice was extended from 4 weeks to 7 weeks. However, class-II-restricted T-cells might still have been activated. Their activation in the Sf.*Cd4^−/−^* mice was likely delayed by the lack of the CD4 co-receptor signal rather than lacking class-II-restricted T-cell response. Treatment with anti-CD4 mAb also delayed the MOI and fatality [21].

### 2.4. *Sf*.Cd28^−/−^ Mice

CD28 on CD4^+^ T-cells interacts with B7 on Ag-presenting cells. This interaction provides the co-stimulation signal required for optimal T-cell activation of CD4^+^ T-cells. Breeding *Cd28^−/−^* mutant gene into Sf mice greatly extended the lifespan of the double mutant mice; 50% of which lived more than 200 days [22]. The spontaneous T-cell activation is greatly reduced in Sf.*Cd28^−/−^* mice as reflected in the presence of a low fraction of CD44^+^ T-cells and the inability of their T-cells upon activation to produce high levels of IFN-γ, IL-4 and IL-10. Paradoxically, IL-2 production upon activation by anti-CD3 and anti-CD28 was comparable among B6, Sf and Sf.*Cd28^−/−^* mice [22]. Serum IgE and IL-4, which were high in Sf mice, were reduced significantly in Sf.*Cd28^−/−^* mice. Consistent with the weakened activation, Sf.*Cd28^−/−^* mice did not show detectable inflammation in liver and lungs [22]. Surprisingly and unfortunately, the effect of *Cd28^−/−^* on skin inflammation was not reported and the perplexing “normal” IL-2 response was not explained [22]. It seems like Sf.*Cd28^−/−^* mice have a generally depressed immune response, such that all MOI were reduced and lifespan prolonged.

### 2.5. *K/BxN*.Foxp3^sf^ Mice

Kouskoff *et al.* generated a B6 TCR Tg mouse line designated KRN mice [23]. Their TCR genes were derived from an RNase-specific B10A.4R T-cell hybridoma. Surprisingly, when KRN mice were crossed with NOD mice, the progeny, *i.e.*, the KRN Tg TCR in {B6xNOD}F1 mice, developed arthritis with severe joint inflammation, which depended on the presence of Tg TCR and Ab with specificity to glucose-6-phosphate isomerase (GPI) [24]. Apparently, a cross-reaction between the Tg TCR and GPI, processed and presented by the I-A^g7^ Ag-presenting cells, initiates the disease process. Negative selection deleted some Tg TCR T-cells in the thymus. Some Tg TCR T-cells (likely expressing dual-TCR) could escape the deletion and emerge in the periphery to initiate the disease process [23]. 

The lifespan of K/BxN.*Foxp3^sf^* mice was prolonged as compared with Sf mice. The arthritis developed faster and was more aggressive as compared with K/BxN mice due to the lack of Treg in the affected joints and an increased production of anti-GPI Ab [25] but the expansion of GPI-specific T-cells was also a likely contributing factor. Like other TCR Tg Sf mice, the majority of CD4^+^ T-cells in the secondary lymphoid organs are CD44^+^. These cells could be expanded both by GPI-specific activation through the Tg TCR or by endogenous TCR of the dual-TCR T-cells [23]. 

### 2.6. *NOD*.Foxp3^sf^ Mice

The NOD.*foxp3^sf^* mice developed more severe MOI but their lifespan was not shortened as compared with B6.*Foxp3^sf^* mice [26]. Their diabetic incidence was not reported, probably because the early death prevented such an analysis [26]. Indeed, the effect of total Treg-deficiency on many chronic autoimmune diseases may not be easily studied because of the dominant and rapid lethality of *Foxp3^sf^* mutation. 

The BDC2.5 TCR Tg specific to an islet Ag was used to generate the BDC2.5/NOD.*Foxp3^sf^* mice to study Treg effect on type-1 diabetes [27]. Both MOI and mortality were ameliorated as compared with Sf or NOD.*Foxp3^sf^* mice. Interestingly, diabetes developed around 2 weeks after birth and 100% incidence was observed at 18 days of age. BDC2.5/NOD.*Rag^−/−^* mice also lacked Treg and developed diabetes earlier than BDC2.5/NOD mice [27]. Diabetes developed at 20 days after birth and 100% incidence occurred at 30 days after birth. The T-cells in BDC2.5/NOD.*Foxp3^sf^* but not BDC2.5/NOD.*Rag^−/−^* mice contain endogenous TCR. The expansion of the dual-TCR T-cells starts around 6 days after birth in Sf mice [16]. The expansion facilitates dual-TCR T-cell participation during diabetes development, if the dual-TCR T-cells are enriched in the islets of BDC2.5/NOD.*Foxp3^sf^* mice. 

### 2.7. *Sf*.Aire^−/−^ Mice

The *Aire* gene is expressed in thymic stromal cells. It controls the synthesis of several tissue-specific Ag required for the deletion of the Ag-specific autoimmune T-cells during thymic selection [28]. Mice with mutant *Aire* genes develop autoimmune diseases directed mostly against endocrine organs [28]. Aire has little influence on Treg expression and function. Sf.*Aire^−/−^* mice have a gravely shortened lifespan even though their endocrine organs remained free from inflammation [26]. 

In these autoimmune models, the Foxp3^Sf^ effect is so dominant that its effect on the specific autoimmune models is difficult to access. It seems that the effect of *Foxp3^sf^* on Aire, NOD, and BDC2.5.NOD mice is largely due to the absence of Treg rather than due to the interactions of the responsible autoimmune gene with the *Foxp3^sf^* mutation.

### 2.8. *Sf*.Il2^−/−^ Mice

IL-2 knockout (*Il2^−/−^)* mice develop lymph node (LN) enlargement and inflammation in colon, liver and salivary glands [12,29]. *Il2^−/−^* mice are deficient in Treg, although a reduced level was present due to compensation from IL-7 and IL-15 and this compensation may have prolonged their lifespan longer than that of Sf mice [30]. The Treg-deficiency may explain the lympho-proliferation and MOI. However, the MOI in Il2−/− mice differs from Sf mice because only the latter develop severe inflammation in skin and lungs [11]. The phenotype of *Il2^−/−^* mice is dominant because Sf.*Il2^−/−^* mice failed to develop skin and lung inflammation whereas their liver inflammation remained [29]. These results strongly suggest that MOI in Sf mice is controlled in an apparent “organ-specific” manner by IL-2.

Interestingly, LN of Sf.*Il2^−/−^* mice are larger and contain more lymphocytes than those in Sf mice. Apparently, lacking IL-2 did not inhibit T-cell activation and proliferation *in vivo*. Other lympho-proliferative cytokines such as IL-4, IL-7, and IL-15 were not higher in Sf.*Il2^−/−^* sera as compared with Sf samples [17]. Reduced FasL (CD178) expression has been implicated in the lymphadenopathy in *Il2^−/−^* mice but FasL expression in Sf.*Il2^−/−^* mice was no less than that in Sf mice [29]. Inhibition of lymphocyte trafficking, either out of LN or into peripheral tissues, or both, may cause accumulation of lymphocytes in the LN. Our recent genome-wide microarray analyses and additional breeding studies on the effect of specific mutant genes on MOI have demonstrated new and heretofore under-appreciated IL-2 functions in the Sf MOI response. This new information will be addressed later in great detail.

### 2.9. *Sf*.Itgae^−/−^ Mice

The control of IL-2 on CD4^+^ T-cell retention in inflamed skin and lungs was demonstrated in Sf and Sf.*Il2^−/−^* mice. Integrinαε (CD103) is a component of a cell surface receptor α_E_β_7_ that binds to E-cadherin expressed mainly by epithelial cells. As a result, T-cells expressing CD103 are retained in tissues like skin and lungs. In Sf mice, the frequency of CD4^+^CD103^+^ T-cells in the LN is significantly higher than the B6 counterpart [31]. An even higher frequency is observed in the skin and lungs. The high frequency of CD4^+^CD103^+^ T-cells is reduced in Sf.*Il2^−/−^* mice, demonstrating the requirement of IL-2 for CD103 expression on CD4^+^ T-cells. *In vitro* culture experiments demonstrated that optimal expression of CD103 also required TGF-β1. IL-2 requirement for CD103 expression is specific for CD4^+^ T-cells including Treg. Interestingly, the Treg deficiency in *Il2^−/−^* mice is observed mainly in the CD103^−^ Treg [25], suggesting a subtle difference in the regulation of CD103 and Foxp3 expression. Perhaps the IL-15- or IL-7-induced Treg does not efficiently express CD103. CD103 expression on CD8^+^ T-cells between Sf and Sf.*Il2^−/−^* mice appears comparable as do their dendritic cells [31].

The inflammation in the skin and lungs but not liver in Sf.*Itgae^−/−^* mice is delayed for a few weeks, indicating that the IL-2-controlled CD103 expression on CD4^+^ T-cells contributed to the “organ-specific” inflammation. However, the lack of CD103 cannot fully explain the complete inhibition of skin and lung inflammation in Sf.*Il2^−/−^* mice because Sf.*Itg*ae*^−/−^* mice eventually develop a severe skin and lung inflammation comparable to that observed in Sf mice. Thus, IL-2 must control additional components of the skin and lung inflammatory process [31].

### 2.10. *Sf*.Fas^lpr/lpr^ Mice

Fas (CD95)/FasL signaling system is known for T-cell homeostasis control but its role in organ damage is less appreciated. As compared with Sf mice, LN lymphocytes in Sf.*Fas^lpr/lpr^* mice increased 20% in number whereas 100% increase was seen for Sf.*Il2^−/−^* mice [29]. The lifespan of Sf.*Fas^lpr/lpr^* mice (12–14 weeks old) was comparable to Sf.*Il2^−/−^* mice. In addition to the skin and lungs, inflammation was extended to other organs. Unlike Sf mice, Sf.*Fas^lpr/lpr^* and Sf.*Il2^−/−^* mice developed inflammation in colon and accessory reproductive organs [12]. These observations suggest that FasL-dependent organ damage is an important factor for mortality induced by the MOI. 

## 3. Participation of Th Subsets in Sf MOI

Th1, Th2, and Th17 cells are known effectors for autoimmune inflammation. Many inflammation conditions including IPEX correlated with enhanced Th2 response and IgE expression, but the contribution by expanded Th1 response was often ignored. The over-emphasis of Th subset imbalance and the frequent attribution of a single Th subset for inflammation often prevent a better understanding of autoimmune regulation. For instance, IL-2 is critically important in Th2 cell development under the Th2 induction condition *in vitro* [32,33], yet its role in allergic inflammation is often ignored by many and rarely addressed [34,35]. The Sf.*Il2^−/−^* mice represent the first animal model in which the role of Th1 cytokine IL-2 in a “Th2-mediated” allergic autoimmune inflammation can be explored. Sf mice displayed highly up-regulated Th1 and Th2 but not Th17 responses. Interestingly, serum IgE, IL-4, IL-5, and IL-13 and CD4^+^ T-cells bearing these cytokines were up-regulated in Sf but not in Sf.*Il2^−/−^* mice as compared with B6 control. This is interesting in light of the fact that the hyper-production of Th2 cytokines (IL-4, IL-5, and IL-13) and IgE in Sf mice were reduced to normal levels in Sf.*Il4^−/−^* mice and yet, skin and lung inflammation persists in the latter group [18]. This comparison suggests that the skin and lung inflammation in Sf mice also involves Th1 responses and that a critical step shared by both Th1 and Th2 responses for skin and lung inflammation must have been inhibited in Sf.*Il2^−/−^* mice.

### 3.1. How IL-2 Controls Skin and Lung Inflammation?

**Figure 1:**
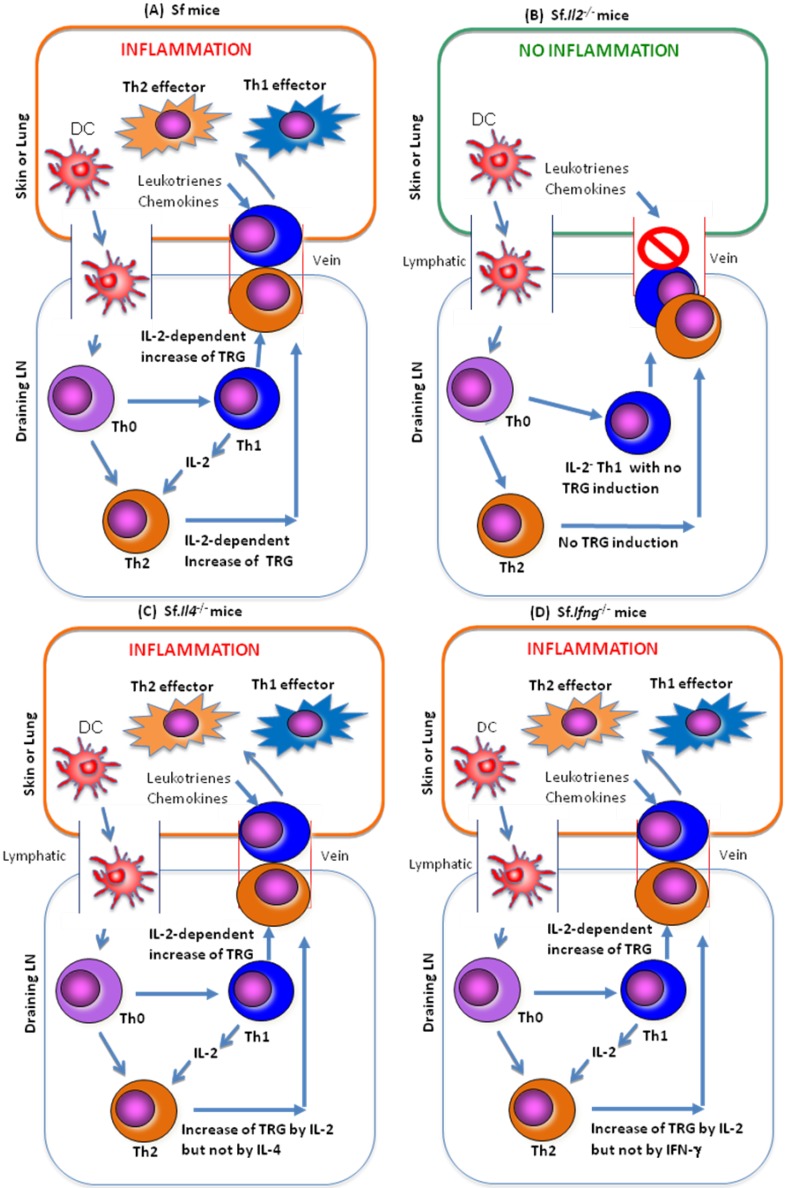
A schematic representation of how IL-2 controls the skin and lung inflammation in Sf mice. (**A**) In the Treg-deficient mice, tissue or environmental Ag are picked up and processed by the dendritic cells (DC), which go to the draining LN and are presented to the Th-cells that have specificity for the Ag. Without Treg and in the presence of IL-2, both Th1 and Th2 responses are expanded. TRG essential for Th cells to go to skin and lungs are induced in both subsets, which then travel to the skin and lungs to induce inflammation; (**B**) In the Sf.*Il2^−/−^* mice, the processed Ag on DC failed to induce a Th2 response due to the absence of IL-2, which is required for Th2 expansion. More importantly, IL-2 is required for the induction of a panel of TRG required for the Th cells to travel to the skin and lungs. IL-2 is not required for the induction of TRG needed for liver inflammation and colitis; (**C**) In the Sf.*Il4^−/−^* mice, the processed Ag on DC induce Th1 response and but the expression of IL-4-, IL-5-, and IL-13-Th2 cells are not expressed or are strongly inhibited. We do not know if they were activated by IL-2. However, the TRG required for skin and lung inflammation are induced in the Th cells by the presence of IL-2. These Th cells are capable of causing skin and lung inflammation; (**D**) In the Sf.*Ifng^−/−^* mice, the processed Ag on DC induced both IL-2-producing Th1 cells and IL-4-producing Th2 cells. Although lacking IFN-γ has a general effect (such as recruitment of leukocytes and enhancing Ag-presentation on inflammation), skin and lung inflammation eventually developed because the IL-2-controlled TRG are induced in the activated Th1 and Th2 subsets.

To address this issue, genes differentially expressed among LN CD4^+^ T-cells of B6, Sf and Sf.*Il2^−/−^* mice were determined [17]. A large number of genes encoding receptors for trafficking, chemotaxis, and retention (altogether abbreviated as the trafficking receptor genes or TRG) were differentially expressed in Sf samples as compared with Sf.*Il2^−/−^* samples. Among them, many skin-homing receptors such as Cysteinyl Leukotriene Receptor 1 (Cysltr1), Leukotriene β4 Receptor 1 (Ltb4r1), CD103, CCR8, and others are the most differentially expressed [17]. These observations suggest that T-cell entrance into skin and lungs is a critical step preceding the T-cell activation in these organs and the subsequent inflammatory response. Consequently, even a strong expansion of potential inflammation-inducing Th subset in the LN cannot induce skin inflammation when the expression of these trafficking receptors is inhibited. The chemotactic factors for T-cell entrance to skin and lungs are likely produced by mast cells, basophils and dermal micro-vessels, melanocytes, and Langerhans cells [36,37]. IL-2, by regulating the receptors for these ligands and others, enables T-cell infiltration into skin and lungs to induce clinical symptoms (Figure 1A–D). 

The most organ-specific autoimmune responses are those mediated by T-cells or Ab that have specificity against the organ-specific Ag. In Sf mice, anti-keratin-14 Abs against skin and anti-pyruvate dehydrogenase-E2 against liver/biliary bile duct have been described [38,39]. However, organ Ag-specific T-cells in Sf mice remain to be established. A selective expansion of organ Ag-specific T-cells by IL-2 is hard to envision. The second control point is at the level of T-cell trafficking that dictates the entrance and long stay of the inflammation-inducing T-cells in the target organs. T-cells that express the receptors for those ligands produced by the target organs and organs that preferentially express ligands for these receptors can display inflammation in an apparent organ-specific manner. Th cytokine-controlled TRG expression allows the entry of T-cells into skin and lungs and the CD103 expression enables longer stay of the CD4^+^ T-cells in the E-cadherin-expressing tissue [17,29]. Similarly, the inflammation in the submandibular gland (SMG) of Sf mice required the production of chemokines induced by toll-like receptor (TLR) agonists [12]. However, *Il2^−/−^* mice develop autoimmune disease, leaving many investigators believing it does not have a “pro-inflammatory” function. The third mechanism is at the stage of T-cell activation in the target organs such as skin and lungs that have a propensity to expand Th2 responses and IgE-mediated inflammation.

### 3.2. Genome-Wide Microarray Comparison among CD4^+^ T-Cells of *Sf* and *Sf*.Il2^−/−^ Mice

To identify the critical targets controlled by IL-2 in the CD4^+^ T-cells, we compared gene expression in the FACS-purified LN CD4^+^ T-cells of Sf and Sf.*Il2^−/−^* mice [17]. An RNA sample prepared from pooled LN CD4^+^ T-cells of two age-matched B6 male were also included for comparison. A total of 346 probes showed significant difference in expression between Sf and Sf.*Il2^−/−^* samples. We eliminated those that were repetitive for the same gene and the genes whose function is not known to be specifically related to the immune system. This maneuver resulted in 79 IL-2-regulated genes that may have a role in the skin and lung inflammation in Sf mice. 

### 3.3. IL-2 Regulates many TRG in the CD4^+^ T-Cells of *Sf* Mice

Among the 79 genes, 38 are well known for their participation in the immune activation and/or inflammatory diseases. Two major differences were observed. First, the over-expression of *Cysltr1* (32-fold), *Ltb4r1* (9-fold), *Il1rl1* (14-fold), *Itgae* (18-fold), and *Ccr1* (8-fold) and to a lesser extent *Itga6* (2-fold), *Ccr2* (2-fold), *Ccr8* (3-fold), and *Cxcr6* (3-fold) was observed in Sf over Sf.*Il2^−/−^* samples. 

The second important observation is that there was no major differential expression of Th cytokine genes involved in inflammation between Sf and Sf.*Il2^−/−^* samples, although many of these genes were over-expressed in both samples when compared with B6 control. It is surprising that many of the Th2 cytokine genes were expressed in Sf.*Il2^−/−^* samples yet these mice lacked skin and lung inflammation. Nevertheless, this interesting observation further supports the importance of up-regulation of TRG on Th cells for organ inflammation.

### 3.4. *Sf* CD4^+^ T-Cells that Displayed Differential Gene Expression Selectively Transferred Skin and Lung Inflammation

To determine whether the organ-specific control of inflammation is an intrinsic property of CD4^+^ T cells, inflammation in organs not dependent on IL-2 was demonstrated [29]. The results showed that CD4^+^ T-cells of Sf but not Sf.*Il2^−/−^* mice induced skin and lung inflammation and the extent of inflammation difference was highly significant. In contrast, comparable levels of inflammation in the liver, pancreas, colon, and SMG were observed in both groups. Moreover, the skin and lung inflammation did not develop throughout the experimental period when the mice became moribund. The study demonstrated that CD4^+^ T-cells that expressed a collective set of IL-2-regulated genes can transfer skin and lung inflammation and that the apparent organ-specificity by IL-2 in Sf mice is an intrinsic property of the CD4^+^ T-cells. 

### 3.5. IL-2 Regulates Expression of Inflammatory Cytokines

As observed in the microarray analyses, many of the inflammatory cytokine genes were highly up-regulated in the CD4^+^ T-cells of Sf and Sf.*Il2^−/−^* mice when compared with B6 CD4^+^ T-cells. It appears that the role of IL-2 in regulating Th2 cytokine gene expression is less impressive than its ability to regulate CD4^+^ T-cell TRG in Sf mice. However, it has been shown in *in vitro* experiments that IL-2 is required for optimal Th2 response [32,33].

To resolve the role of IL-2 in Th cytokine production *in vivo*, we determined the serum levels of these cytokines by multiplex cytokine assay even though some of those are also produced by non-CD4^+^ T cells, and this measurement represents the cumulative expression of the cytokines [17]. We observed no difference in the expression of TNF-α or IFN-γ between Sf and Sf.*Il2^−/−^* mice. Surprisingly, IL-4, IL-5, and IL-13 were significantly lower in the Sf.*Il2^−/−^* sera. IL-3 and M-CSF were also markedly lower in Sf.*Il2^−/−^* sera. Serum IL-10 and IL-17 were not significantly different between Sf and Sf.*Il2^−/−^* mice [17]. 

We also conducted *ex vivo* stimulation of the CD4^+^ T-cells from B6, Sf, and Sf.*Il2^−/−^* mice to determine whether the specific Th cytokine-producing cells were differentially regulated during T-cell activation. The results showed that the frequency of Th2 but not Th1-cells was significantly lower in Sf.*Il2^−/−^* CD4^+^ T-cells as compared with Sf samples [17]. 

Our study suggests that IL-2 regulates skin and lung inflammation at two different stages of the inflammation process. The major targets of IL-2 in Sf mice are those receptors required for CD4^+^ T-cell trafficking. IL-2 also controls the cumulative levels of Th2 cytokines in Sf mice and the frequency of Th2 cells during T-cell activation. Our results suggest that the differentially displayed genes induced by and during T-cell activation play a critical role in this process. 

### 3.6. Restoration of TRG Expression by rIL-2

To determine whether IL-2 can restore TRG expression, we stimulated the FACS-sorted CD4^+^ T-cells from 15 days old B6 male, Sf, and Sf.*Il2^−/−^* mice in the presence of exogenous rIL-2 for 3 days. Both IL-2 and IL-4 were able to restore the expression of *Cysltr1*. In this case, IL-2 could have induced IL-4, which then induced *Cysltr1* expression, an indirect pathway that could occur for other IL-2-regulated genes. A weak trend of increase of *Il1rl1* by IL-2 was noted in Sf.*Il2^−/−^* samples. This is similar to the partial restoration of CD103 by IL-2 [29]. The *Ltb4r1*, *Il1rl1*, and *Ccr1* genes, which were expressed at lower levels in the Sf.*Il2^−/−^* mice, could not be restored to the level in Sf mice by the 3-day stimulation. Because the expression of *Itgae* was regulated both by TGF-β1 and IL-2, we also added rTGF-β1 to the culture system along with IL-2. However, the combination was unable to restore the expression of *Ltb4r1*, *Il1rl1*, and *Ccr1* on the CD4^+^ T-cells of Sf.*Il2^−/−^* mice. Interestingly, the expression of *Ltb4r1*, *Il1rl1*, and *Ccr1* in the CD4^+^ T-cells from the Sf mice was inhibited when rTGF-β1 was present. TGF-β1 also inhibited the IL-4 induction of *Cysltr1* in the Sf.*Il2^−/−^* sample. Thus, the inability of IL-2 or IL-4 to restore TRG expression in Sf.*Il2^−/−^* CD4^+^ T-cells *in vitro* is not due to lack of TGF-β1. This could be the result of an irreversible differentiation of the CD4^+^ T-cells due to the repeated *in vivo* stimulation of the cells in the absence of IL-2. Thus, at least two pathways are used by IL-2 to regulate TRG expression. Those that can be completely or partially restored by IL-2 are likely under the direct control of IL-2 signal for gene activation and those that cannot be immediately restored appear to require additional cell differentiation processes or other cytokines for their up-regulation *in vivo*. 

### 3.7. Th1 Response is Dominant and Controlling in Skin and Lung Inflammation in *Sf* Mice

Th1 response is defined by its ability to produce IL-2 and IFN-γ, although IL-2 production is transient as compared with IFN-γ. Our study with Sf.*Il2^−/−^* mice, therefore, indicates the dominant and controlling Th1 response to the skin and lung inflammation in Sf mice. Because IL-2 and IFN-γ control different aspects of the inflammatory responses, we compared the autoimmune response between Sf.*Ifng^−/−^* with Sf.*Il2^−/−^* mice.

### 3.8. MOI in *Sf.*Ifng^−/−^ Mice

IFN-γ is the principal marker for Th1 *versus* Th2 cells, but NK and CD8^+^ T-cells also produce high amounts of IFN-γ. As a marker, the absence of IFN-γ, particularly in knockout mutant strains such as *Ifng^−/−^* or *Tbx21^−/−^* [40,41], does not mean they lack Th1-cells. IFN-γ inhibits Th2 response under the *in vitro* induction condition skewed against Th2 development. It activates macrophages, NK cells and neutrophils, particularly in the presence of LPS, to become potent inflammatory cells. It induces CXCL9, CXCL10, and CXCL11 from various cells to attract leukocytes to target organs [42,43]. It induces strong MHC expression and as such exacerbates ongoing immune response. Less is known for its effect on TRG regulation in CD4^+^ T-cells. Because both Sf.*Il2^−/−^* and Sf mice had high serum IFN-γ and IFN-γ^+^ CD4^+^ T-cells [12], yet only the Sf mice developed inflammation in the skin and lungs, the question as to what extent the IFN-γ influenced the inflammation was addressed. 

Breeding *Ifng^−/−^* mutant gene into Sf mice decreased the cytokine response of CD4^+^ T-cells that produced IL-2, TNF-α, IL-4, IL-5, and IL-13. Because a large fraction of IFN-γ is produced by NK and CD8^+^ T-cells and IFN-γ has a very different inflammation-inducing function from IL-2, different manifestations of inflammation occur. The clinical signs of inflammation in the skin, eyes, ears and tail were reduced and delayed by 1–3 weeks. The inflammation in ears, skin, lungs and liver in the 3 weeks old Sf.*Ifng^−/−^* mice was still statistically significantly developed when compared with B6 controls. The lifespan of Sf.*Ifng^−/−^* mice was prolonged to 6–7 weeks and the MOI was fully developed at that time. Surprisingly, the total number of CD3^+^, CD4^+^ T-cells and total lymphocytes in the LN (3 weeks old) were comparable to Sf samples (3 weeks old). The results are in contrast to Sf.*Il2^−/−^* mice in which the inflammation in the skin and lungs was inhibited for the entire lifespan even in the presence of increased IFN-γ and expansion of lymphocytes. It is important to note that absence of IFN-γ does not mean the affected response is mediated by Th1 response unless transfer by IFN-γ^−/−^ Th cells induced the disease in adoptive transfer experiments.

### 3.9. *Sf*.II4^−/−^ and *Sf*.Stat6^−/−^ Mice Develop Inflammation in the Skin and Lungs

In the Sf.*Il4^−/−^* and Sf.*Stat6^−/−^* mice, the clinical signs of skin inflammation and lethargy appear similar to Sf mice [18]. Histological analysis reveals strong inflammation in the skin, lungs and liver in these mice even though their Th2 response based on IL-4 production was totally inhibited in Sf.*Il4^−/−^* mice or greatly reduced in Sf.*Stat6^−/−^* mice. The total lymphocytes were not different among Sf, Sf.*Il4^−/−^* and Sf.*Stat6^−/−^* mice. In contrast, inflammation in the skin and lungs but not liver was inhibited in Sf.*Il2^−/−^* mice even though the total LN lymphocytes were significantly higher than Sf mice. Thus, IL-4/STAT6-dependent response was not required for the skin and lung inflammation in Sf mice. 

## 4. Comparison of Cytokine-Producing Profiles of CD4^+^ T-Cells

### 4.1. Cytokine-Producing CD4^+^ T-Cells upon ex vivo Activation

The LN CD4^+^ T-cells that produced IL-2, IFN-γ, TNF-α, IL-10, IL-4, IL-5, IL-13, and IL-17 were compared among 3-week old B6, Sf, Sf.*Il2^−/−^*, and Sf.*Il4^−/−^* mice upon *ex vivo* activation [18]. B6 mice expressed few IFN-γ^+^CD4^+^ T-cells, which were increased significantly in Sf mice. In Sf.*Il2^−/−^* mice, IL-2^+^CD4^+^ T-cells were absent but the frequency of IFN-γ^+^CD4^+^ T-cells was similar to Sf mice, indicating that IL-2 deficiency did not affect IFN-γ production in Th1 cells. 

B6 mice had few IL-4^+^CD4^+^, IL-5^+^CD4^+^, and IL-13^+^CD4^+^ T-cells, which were significantly increased in Sf mice. The expression of these CD4^+^ T-cells was strongly inhibited in Sf.*Il2^−/−^* mice. In Sf.*Il4^−/−^* mice, IL-4^+^CD4^+^ T-cells were not detected. IL-5^+^CD4^+^ T-cells were strongly inhibited, and a significant but moderate inhibition was observed for IL-13^+^CD4^+^ T-cells. The IFN-γ^+^CD4^+^ T-cells (~60%) in Sf.*Il4^−/−^* mice were significantly higher than that in Sf and Sf.*Il2^−/−^* mice. In addition, this value was higher than the 30% of IL-2^+^CD4^+^ T-cells in the same mice. This could be caused by the absence of negative regulation of IFN-γ production by STAT6 [44]. This increase could compensate for the reduction of Th2-mediated inflammation in Sf.*Il4^−/−^* and Sf.*Stat6^−/−^* mice in the skin and lungs. By contrast, a significant expression of IL-10^+^CD4^+^ T-cells was observed in Sf.*Il4^−/−^* and Sf.*Il2^−/−^* samples, suggesting IL-10 expression in Sf CD4^+^ T-cells is not controlled by IL-2 and IL-4. Similarly, the frequency of TNF-α^+^CD4^+^ T-cells was also increased in Sf mice as compared to B6 control and the strong expression was not diminished in Sf.*Il2^−/−^* and Sf.*Il4^−/−^* samples. 

In contrast to Sf.*Il4^−/−^* mice, IL-4^+^ Th2 cells were observed in Sf.*Stat6^−/−^* mice. Although IL-4^+^ Th2 cells were significantly reduced as compared with Sf samples, they were still significantly higher than B6 samples [18]. This suggests that IL-2 is more critical than STAT6 in regulating the development of IL-4^+^ Th2 cells but STAT6 is still needed for the optimal expansion of the IL-4^+^CD4^+^ T-cells in Sf mice.

Th17 cells are an important effector Th subset in certain autoimmune diseases but how pervasive and the contribution of this subset to Sf skin and lung inflammation in Sf mice has not been determined. In Sf mice, despite losing Treg and developing severe MOI, Th17 cells were few as compared with Th1 and Th2 cells [18]. This low value was maintained in all Sf double mutants examined. Th17 cells express IL-10R and Th17 expansion could be inhibited by IL-10 produced by non-Treg [45]. In summary, the cytokine expression profiles of Th subsets indicate that IL-2 is the major cytokine critical to the development of skin and lung inflammation in Sf mice.

### 4.2. Serum Levels of Cytokines and IgE do not Always Reflect Inflammation Status in the Skin and Lungs of *Sf* and *Sf* Double Mutants

Serum levels of various cytokines (IL-2, IL-4, IL-5, IL-6, IL-10, IL-13, IL-17, IFN-γ, and TNF-α) and IgE of age-matched mice were determined [18]. Sera from Sf mice contained high levels of cytokines associated with Th1 and Th2 responses. Great variability was observed because some cytokines were also produced by non-Th cells during inflammation. Low expression of IL-4, IL-5, and IL-13 was observed in Sf.*Il2^-/-^* sera. The only cytokine that was low and not significantly increased was IL-17 in all samples tested. Our studies suggest that the most influential cytokine for skin and lung inflammation correlates with serum IL-2. Other cytokines had only partial and fractional effects on specific aspects of an inflammation response.

The serum IgE level was dramatically increased in Sf mice as compared with B6 sera. It was inhibited to undetectable levels in the Sf.*Il4^−/−^* and Sf.*Stat6^−/−^* mice whereas it remained high in Sf.*Il2^−/−^* and Sf.*Ifng^−/−^* mice. Interestingly, Sf.*Il2^−/−^* mice expressed a significant serum level of IgE probably due to IL-4 expression by non-Th2 cells [18]. These data demonstrate that IgE is not essential for the inflammation in the skin and lungs in the Sf and the Sf double mutant mice examined herein. 

## 5. Th Cytokines Regulate TRG: Mechanism and Specificity

### 5.1. IL-2 but not IL-4 or IFN-γ Regulates TRG for Skin and Lung Inflammtion

IL-2 regulates not only the Th2 response but also TRG in CD4^+^ T-cells that are capable of transferring inflammation to the skin and lungs of *Rag1^−/−^* recipients [12]. Therefore, it becomes important to determine whether the over-expression of these genes is also restricted to a specific Th subset or regulated by a specific Th cytokine.

The expression of several TRG in the CD4^+^ T-cells that had been implicated in the inflammation of skin and lungs was determined by quantitative PCR [18]. In Sf.*Il4^−/^*^−^ mice, the marked increase in *Cysltr1*, *Ltb4r1*, *and Il1rl1* was not observed in CD4^+^ T-cells but the inflammation in the skin and lungs remained. The IL-4-dependent expression of these genes was confirmed with the results from Sf.*Stat6^−/−^* samples. The TRG selected for examination were: *Cysltr1*, *Ltb4r1*, *Ptgir*, *Il1rl1*, *Ccr1*, *Ccr3*, *Ccr4*, *Ccr8*, *Cxcr3 and Cxcr6*, chosen because some of them were selectively increased in Sf CD4^+^ T-cells as compared with Sf.*Il2^−/−^* samples whereas others were enhanced in both samples as compared with B6 CD4^+^ T-cells [12]. The expression of *Cysltr1*, *Ltb4r1*, and *Ptgir* were significantly inhibited in Sf.*Stat6^−/−^* mice as compared with Sf mice. The expression of *Il1rl1* was also reduced, but was not statistically significant [18]. In contrast, the expression of *Ccr1*, *Ccr3*, *Ccr4*, *Ccr8*, *Cxcr3* and *Cxcr6* remained high in Sf.*Stat6^−/−^* mice. These observations indicate that the *Cysltr1*, *Ltb4r1*, *Ptgir*, and perhaps *Il1rl1* genes are preferentially regulated by IL-4/STAT6 and that the Th1 cells do not need their expression to induce inflammation in the skin and lungs in the Sf mice. 

Our studies showed that when compared with B6 CD4^+^ T-cells, *Ccr1* and *Ccr8* were selectively enhanced in Sf but not Sf.*Il2^−/−^* mice whereas *Ccr3*, *Ccr4*, *Cxcr3 and Cxcr6* were up-regulated in both mice [12]. The data obtained with Sf.*Ifng^−/−^* and Sf.*Stat6^−/−^* mice demonstrated that *Cxcr3* expression among the genes examined was critically dependent on IFN-γ [18]. The Sf.*Stat6^−/−^* mice had the highest IFN-γ and this correlated with the strongest expression of *Cxcr3* and *Cxcr6* among all samples examined. Overall, these data demonstrate that inhibiting the TRG regulated by IL-4/STAT6 or IFN-γ is not sufficient to prevent inflammation in the skin and lungs. 

Thus, Th responses that control TRG could be summarized as follows. One set of TRG (*Cysltr1*, *Ltb4r1*, *Il1rl1*, *Ptgir* and *Ccr4*) is regulated by IL-2 through the IL-4 produced by Th2 response. These TRG were inhibited in Sf.*Il4^−/−^* mice but not inhibited in CD4^+^ T-cells of the Sf.*Ifng^−/−^* mice and would be important in the skin and lung inflammation that develop late in the Sf.*Ifng^−/−^* mice. The other set of TRG (*Ccr1*, *Ccr3*, *Ccr8*, *Cxcr3 and Cxcr6*) are the ones that are independent from the Th2 response and are regulated by the Th1 cytokines. These TRG are responsible for the skin and lung inflammation in the Sf.*Il4^−/−^* mice. Because IL-2 is needed for TRG of Th1 and Th2 cells, it has a more dominant role than Th2 IL-4 in regulating skin and lung inflammation in the Sf mice. A scheme that summarizes the roles of IL-2, IL-4, and IFN-γ in skin and lung inflammation in Sf mice is presented in Figure 1.

It is important to note that there are up-regulated TRG in Sf, Sf.*Il2^−/−^*, Sf.*Ifng^−/−^* and Sf.*Il4^−/−^* Th-cells and these mice displayed liver inflammation and colitis. At present, we have not attempted to figure out what are the critical TRG for the entrance of the inflammation-inducing Th-cells into liver and colon. 

### 5.2. Study of *Sf*.Ltb4r1^−/−^, *Sf*.Alox5^−/−^, *Sf*.Cx3cr1^gfp/gfp^ and *Sf*.Il10^−/−^ Mice

This series of studies included Sf double mutant mice from which additional information were obtained with respect to the effect of cytokines and TRG on Th subset expression and MOI, respectively [18].

The Sf.*Ltb4r1^−/−^* mice had expanded Th1 and Th2 responses and inflammation in ears, skin, lungs and liver. With only 4 mice examined, there was some variability in histological scores for ears, skin and lungs. Two mice died at 24 days and the other two died at 31 days. On the other hand, Sf.*Alox5^−/−^* mice displayed severe MOI. Their Th1 cells did not change much but their Th2 cells were twice as many as that in the Sf mice, and they died within 3 weeks after birth. This could be due to the fact that 5-lipoxygenase produces pro-inflammatory leukotrienes that target Th2 cells, neutrophils, monocytes, and macrophages [46]. Collectively, the results indicate that leukotrienes and leukotriene receptors could contribute to but are not as critical as IL-2 to the MOI in Sf mice. 

The *Cx3cr1* gene encodes Cx3cr1 in lymphocytes, monocytes and macrophages [29]. Its deletion in Sf.*Cx3cr1^gfp/gfp^* mice had no effect on the MOI, consistent with the lack of *Cx3cr1* up-regulation in Sf CD4^+^ T-cells as compared with B6 samples [12]. The MOI was not inhibited in Sf.*Il10^−/−^* mice, indicating that IL-10 had little effect on the Sf autoimmune manifestation, most likely due to the expression of multiple inflammatory effector mechanisms [12]. 

In summary, these studies clearly demonstrate that many of the commonly considered elements of inflammation processes appear unable to influence the spontaneous inflammation in Sf mice. This is most likely due to the fact that this fatal autoimmune disease has multiple components and pathways for inflammation and that eliminating a single component is usually non-effective in preventing the development of inflammation. In this regard, it is not surprising that without Th2 response, IgE, IFN-γ, the skin and lung inflammation still develop due to participating Th1 response. It just happens that IL-2 is required for the induction of TRG for Th cells trafficking to skin and lungs and we discovered this new function of IL-2 by comparing Sf and Sf.*Il2^−/−^* mice and demonstrated that Sf.*Il2^−/−^* mice lack these TRG and do not develop skin and lung inflammation.

## 6. Environmental and Age Effects on MOI

The Sf mice display the most severe form of MOI, but the early death prevents other potential organ inflammations that may not have had a chance to develop. There are several reasons why inflammation in these organs develops later than skin, lungs and liver and efforts to understand these reasons from environment, aging, and organ development will be addressed here.

Skin, lungs, gastrointestinal system, and liver are the first large-size organs exposed to self and foreign Ag. The skin and lungs are exposed to Ag floating in the air and the skin is further exposed to areas of contacts such as bedding. Liver and gastrointestinal organs are exposed to food Ag from mother’s milk. The mother milk contains IgA that would protect against microbiota expansion and reduce the stimuli in the gastrointestinal tract. These may account for the early and delayed manifestations of inflammation in these organs.

As discussed earlier, Fas mutation prolongs the lifespan of Sf mice from 4 weeks to 12–17 weeks old and this effect seems to be largely due to FasL-mediated organ damage [12]. Nevertheless, the delayed death allows the development of new autoimmune inflammation in other organs not previously observed in Sf mice. Subsequently, other approaches such as that of Sf.*Itgae^−/−^* mice were developed that both validated the study of Sf.*Fas^lpr/lpr^* mice and facilitated a deeper understanding of environment and age effects on autoimmune response in Sf mice.

Critically speaking, the MOI in Sf mice cannot be considered an autoimmune response without the identification of the target Ag and their organ-specific association. Perhaps the best example is the gastritis induced by day-3-thymectomy that activates both T and B cell responses against the H^+^/K^+^-ATPase of stomach parietal cells [47,48]. In experimental autoimmune prostatitis and oocytis, specific responses against EAPA and MATER organ Ag have been implicated [49,50]. Ab against a mitochondrial Ag associated with cholangitis has been demonstrated in Sf mice [39]. Because Treg controls immune responses to both self and foreign Ag, it is possible that the MOI is also contributed by foreign Ag that are more often associated with a particular organ in the host including the steady presence of Ag from the environment, food, bedding, microbiota, and growth changes [12,51].

Sf mice die around 24–28 days old with severe inflammation in the ear, conjunctiva, skin, lungs, liver and tail. Common autoimmune diseases such as thyroiditis, diabetes, encephalomyelitis, arthritis, glomerulonephritis and inflammation in the oral and gastrointestinal tracts are not observed [26,52]. To determine if early death or pre-weaning conditions prevented the expression of inflammation in these organs, *Fas^lpr^*^/*lpr*^ gene was bred into Sf mice to prolong the lifespan to 8–20 weeks. Using this approach, additional inflammation was observed in colon and accessory reproductive organs [8]. Thus, pre-weaning conditions may not be the only factor that affects organ-specific inflammation. Another method was by transferring Sf LN cells into *Rag1*^−/−^ recipients [52]. This approach not only induced severe inflammation in the skin, lung and liver but also in salivary gland, stomach, pancreas, small intestine, and colon, perhaps due to long lifespan and environmental changes. A summary figure showing the MOI under various conditions was presented in reference11.

### 6.1. Skin Inflammation

Skin inflammation is the earliest external symptom observed in Sf mice. The severely inflamed areas are ear, eyelids, and tail. Conjunctivitis is probably worsened by frequent scratching surrounding the ears and eyes. Adoptive transfer of Sf LN cells into adult *Rag1*^−/−^ recipients induced skin inflammation first in the eyelids. In contrast, tail inflammation is minimal, suggesting organ development control of tail inflammation. Sf skin inflammation coincided with a strong Th2 response and high serum IgE [53] but the Th2 cytokines and IgE expression occurred together with a strong Th1 type response and that strong skin inflammation was observed in Sf.*Il4*^−/−^ mice [18]. In contrast, skin inflammation in Sf.*Il2*^−/−^ never developed and their LN cells also failed to induce skin inflammation upon transfer into *Rag1*^−/−^ recipients.

### 6.2. Inflammation in Salivary and Lacrimal Glands

Sjögren's syndrome is characterized by inflammation in the salivary glands and lacrimal glands with dry mouth and dry eyes. *Il2*^−/−^ and *Il2rα*^−/−^ mice develop inflammation in these glands and their ability to produce saliva upon stimulation with Pilocarpine is impaired [54]. Interestingly, Sf mice do not develop inflammation in these glands but transfer of Sf LN cells into *Rag1*^−/−^ recipients induced strong inflammation in these organs [54]. The SMG is the major mouse saliva-producing organ and its development is age-dependent and sexually dimorphic [55]. The acini of the SMG develop soon after birth and dominate in the early phase of SMG development. The granular convoluted tubules (GCT) develop around 3–4 weeks of age and the expression is markedly stronger in male than female. Infiltration in the salivary glands in *Il2*^−/−^ mice as well as *Rag1*^−/−^ recipients of Sf LN cells was observed primarily in the areas of acini but also noticeable around the GCT areas, with destruction and disappearance of the acini and atrophy of GCT [55]. The SMG in Sf mice was not only free from inflammation but also growth-arrested in that the male-dominant expression of GCT was inhibited, leaving the organ mostly occupied by the acini. Several observations suggest that GCT development is not important for SMG inflammation. First, treatment of Sf.*Fas^lpr^*^/*lpr*^ mice with testosterone fully restored GCT development but failed to induce inflammation in the SMG. Second, SMG inflammation was observed in *Il2*^−/−^ mice that also have a greatly reduced GCT expression (but not as severe as Sf mice). Third, treatment of Sf mice with daily oral application of LPS or Poly:I/C induced SMG inflammation in the absence of GCT development [12]. The latter observations suggest that defect in innate immunity and Ag-presentation may be involved during the development of SMG.

### 6.3. Lung Inflammation

Similar to skin, lungs are constantly exposed to environmental Ag. Lung inflammation in Sf mice is characterized by the severe infiltration of leukocytes around the bronchia and alveoli. In contrast, lung inflammation was not observed in *Il2*^−/−^ mice and Sf.*Il2*^−/−^ mice [29]. A recent study has demonstrated that lacking IL-10-producing Treg is inductive to skin and colon inflammation [56]. However, a normal level of IL-10 mRNA expression was observed in the Treg of *Il2*^−/−^ mice [57].

In contrast to *Il2*^−/−^ mice, *Il2rα*^−/−^ mice display severe lung inflammation. *Il2rα*^−/−^ mice differ from *Il2*^−/−^ mice in that they over-express CD8^+^ memory T-cells that occupy more than 75% of the total T-cell repertoire. As a result of lacking high affinity IL-2R, *Il2rα*^−/−^ mice accumulated high serum levels of IL-2 which could stimulate through the low-affinity IL-2R on CD8^+^ T-cells (un-stimulated CD4^+^ T-cells express few low-affinity IL-2R) [58]. It is tempting to speculate that these CD8^+^ T cells are responding to air and environmental Ag such as virus that are presented through class-I Ag processing pathway by the lung Ag-presenting cells.

### 6.4. Gastritis and Small Intestine Inflammation

Gastritis and small intestine inflammation are neither observed in Sf mice nor in the Sf.*Fas^lpr^*^/*lpr*^ mice that have a prolonged lifespan beyond weaning. However, they were induced by transfer of Sf LN cells into *Rag1*^−/−^ recipients [52]. Moreover, gastritis and small intestine inflammation were not observed in *Il2*^−/−^ mice and transfer of Sf.*Il2*^−/−^ LN cells failed to induce inflammation in the organs. Thus, adoptive transfer of LN cells from Sf and Sf.*Fas^lpr^*^/*lpr*^ mice into *Rag1*^−/−^ recipients remains the only protocol capable of inducing gastritis and small intestine inflammation. Although we observed inflammation around the areas containing the parietal cells, whether this inflammation includes a component against H^+^/K^+^ ATPase, like the case in the BALB/c mice thymectomized at 3 days after birth, is unknown at present.

### 6.5. Liver Inflammation and Cholangitis

Both *Il2*^−/−^ and Sf mice develop liver inflammation manifested by peri-vascular infiltration of leukocytes. High titers of Ab against pyruvate dehydrogenase complex component E2 characteristically associated with cholangitis were identified in the sera of Sf mice [52]. Leukocyte infiltration was observed around portal areas with damage in the biliary duct. Livers from Sf mice strongly expressed inflammatory cytokines including TNF-α, IFN-γ, IL-6, IL-12 and IL-23 [52]. Presence of anti-E2 Ab was also detected in *Il2*^−/−^ and Sf.*Il2*^−/−^ mice. Autoimmune cholangitis was observed in *Il2Rα*^−/−^ mice and it was shown that this autoimmune disease is, in contrast to colitis, more dependent on the expression of CD8^+^ T-cells [58,59].

### 6.6. Pancreatitis

Although often associated with IPEX patients [60], Type-1 diabetes was neither observed in Sf mice nor in *Rag1*^−/−^ recipients of Sf LN cells. Sf, Sf.*Fas^lpr^*^/*lpr*^, Sf.*Il2*^−/−^ or adult *Rag1*^−/−^ recipients of Sf LN cells developed very mild pancreatitis with peri-vascular infiltration of leukocytes and occasional destruction of acini outside the islets. Interestingly, moderate to severe pancreatitis with strong leukocyte infiltration and severe destruction of acini was observed when Sf LN cells were transferred IP into neonatal or adult *Rag1*^−/−^ recipients [52]. Using Sf.*OT-II* mice, we showed that in the absence of Treg, Ag-reactive T-cells still required the Ag in order to expand, *i.e.*, OT-II clonotypic T-cells were not expanded due to the absence of OVA whereas dual TCR T-cells were expanded by the host Ag [16]. Thus, Treg-deficiency plays more of a facilitating role in the disease process. As type-1 diabetes is often observed in IPEX patients, this study suggests that the IPEX patients must have naturally occurring auto-reactive T-cells with sufficient binding activity against the islet components present in their system.

### 6.7. Colitis

Sf mice do not develop colitis and this is likely due to the weaning condition because Sf.*Fas^lpr^*^/*lpr*^ mice that lived longer than 4 weeks begin to develop colitis. Moreover, transfer of Sf LN cells into adult *Rag1*^−/−^ recipients also induced colitis. Transfer into neonate *Rag1*^−/−^ recipients induced little inflammation in the colon before weaning but severe colitis rapidly developed afterward [52]. *Il2*^−/−^ mice also develop colitis but only after weaning. It is likely that the microbiota present in the colon contributes to the colitis development. Colon seems to have the easiest millieu for inflammation induced by adoptive transfer of effector T-cells. Even T-cells expressing a single transduced TCR often induce colitis and this system has been used to address the “auto-reactive” repertoire of Treg [61]. It is not clear why such T-cells often selectively induce colitis but not inflammation in other organs, had they had specificity against a self Ag. The possibility that these TCR recognize food antigens and colon microbiota has been raised [62]. Moreover, the constant activation of cells of innate immunity and the high local levels of type-I interferon and IL-1b may be contributors as well.

Integrin α_E_(CD103)β7 is a critical homing and retention receptor for lymphocytes traveling to and lodging in E-cadherin-expressing organs., But CD103^−^CD45RB^high^ cells have been shown to induce colitis upon transfer [63]. We generated Sf.*Itgae*^−/−^ mice and they had a prolonged lifespan of 7–8 weeks old. They developed colitis and their LN cells were able to transfer colitis to *Rag1*^−/−^ recipients, demonstrating the presence of a CD103-independent mechanism for colitis [31].

### 6.8. Myositis

Sf mice do not develop inflammation in the skeletal muscle but direct injection of Sf LN cells into the limb muscle of *Rag1*^−/−^ mice induces inflammation not only in the injected sites but also in the skeletal muscle of the other limbs [52]. In contrast, intravenous transfer of Sf LN cells failed to induce skeletal muscle inflammation. It is possible that directly injected T-cells first induced damage of muscle cells and the released Ag were recognized by the specific T-cells present in the LN cells and expanded. How these T cells travel through the circulation to induce inflammation in other limb muscle remains to be established. 

### 6.9. Inflammation in the Accessory Reproductive Organs

Although Sf.*Fas^lpr^*^/*lpr*^ mice have a prolonged lifespan beyond the adult age, they remain reproductively incompetent with low body weight and under-developed reproductive organs. Gross examination revealed a tremendous atrophy in the coagulation glands/seminal vesicle, preputial glands, epididymis and prostate. Histological examination confirmed the atrophy of these organs, displayed as shrunken glands and empty lumens. This was accompanied by a prominent presence of leukocyte infiltrates in the peri-glandular regions, in the interstitial regions of the testis, and in the regions containing the interstitial Leydig cells between the seminiferous tubules. Testosterone treatment successfully restored the growth of the accessory reproductive organs of Sf.*Fas^lpr^*^/*lpr*^ mice but the leukocyte infiltrates in the reproductive organs were still present [14].

By breeding and adoptive transfer experiments under various experimental conditions, Sf mice are shown to contain a large repertoire of T-cells capable of inducing inflammation in a large number of organs and tissues. Polyclonal Treg can suppress the MOI, suggesting the presence of a large repertoire of functional Treg as well [52]. An important issue is to determine whether inflammation in individual organs is organ-specific and what they are specific to. In this regard, Sf mice should be useful to identify organ-specific autoimmune T-cells and their Treg counterparts, although this remains a daunting task. 

## 7. Conclusions

As a result of the Foxp3 mutation and absence of functional Treg, Sf mice develop fatal MOI within 28 days after birth. This rapid MOI and experimental manipulations can be exploited to further explore the mechanisms, specificities, and consequences of the MOI. We discussed the biology of Sf MOI from the points of views of genetic, cellular, cytokine, molecular, environment, and age aspects. Table 1 summarizes all the available information from Sf double mutant mice available from the literature and our own studies. Amongst these studies, the most surprising and important finding is the discovery of the previously unrecognized novel role of IL-2 in the expansion of TRG in Th1 and Th2 cells and Th2 cytokine production. Both TRG expression and Th2 response are involved in the skin and lung inflammation in Sf mice. In addition, the mechanisms and consequences of some of the affected genes were addressed. While the study of Sf mice can be efficient in order to understand the biology of MOI, we believe that the genetic control of MOI is similar to cancer in that without correcting the genetic defect, the disease cannot be easily cured. This is the major reason why in our series of studies various factors that are well known for participation in skin and lung inflammation such as IgE, Th2 cytokines, and IFN-γ either cannot inhibit or delay the inflammation. It appears that the genetic defect for inflammation needs a genetic correction for curing the disease. 

**Table 1:** Changes of lymphocyte subsets, MOI, and lifespan of various Sf double mutant mice.

Gene examined	Change in lymphocytes	Change in MOI	Lifespan
Sf [21]	Th1 and Th2 subset expansion	MOI in skin, lungs and liver	3–4 wk
Sf*.TgTCR.Rag*^−/−^ [13]	TCR Tg T-cells only	No MOI	>20 wk
Sf*.Cd4*^−/−^ [21]	No CD4^+^ T-cells	Delayed 1 wk	6 wk
Sf*.β2m*^−/−^ [21]	No CD8^+^ T-cells	Not delayed	4 wk
Sf.*TCR Tg* [13]	T-cells reduced, dual-TCR T-cells expanded	Delayed 2–3 wk	7 wk
Sf.NOD [26]	N.D. *	More severe than B6.*Foxp3^sf^* mice	N.D.
Sf.*BDC2.5 Tg TCR* in NOD [27]	Lympho-proliferation was ameliorated	Rapid development of insulitis and diabetes, MOI was not addressed.	N.D. **
Sf*.Aire*^−/−^ [28]	N.D.	MOI fastened but did not extend to endocrine organs.	2–3 wk
Sf*.Cd28*^−/−^ [22]	Inhibit T-cell activation and cytokine production	Inhibited	50% lived >30 wk
Sf*.Stat6*^−/−^ mice (Balb/c) [53]	Inhibit IgE and Th2 cytokine production	Inhibited eosinophilia and lung Goblet cell metaplasia	5 wk
Sf*.Fas^lpr/lpr^* [12,29]	Slight increase in lymphocytes in LN	Not delayed but lifespan prolonged, developed inflammation in accessory reproductive organs and colitis	6–18 wk
Sf*.Itgαε*^−/−^ [31]	Lymphocyte number decreased by ~40%	Delayed 2–3 wk, developed colitis	6–7 wk
Sf.*Il2*^−/−^ [29]	Lymphocytes in LN increased 100%. CD103 and trafficking receptors inhibited	Delayed 3–5 wk. No skin inflammation, greatly reduced lung inflammation, liver inflammation remained, developed colitis	6–10 wk
Sf.*Il4^−/−^* [18]	IL-4, IL-5, and IL-13 CD4^+^ T-cells were inhibited. TRG controlled by IL-4 were inhibited. IgE expression was inhibited	Skin and lung inflammation were not inhibited	4 wk
Sf*.Stat6^−/−^* (B6) [18]	Reduced IL-4, IL-5 and IL-13 CD4^+^ T-cell expression. TRG controlled by Stat6 were inhibited. IgE expression was inhibited. TRG controlled by IL-2 were not affected	Skin and lung inflammation were not inhibited	4 wk
Sf*.Ifng^−/−^* [18]	Lymphocyte expansion delayed but fully restored later. IL-2-producing Th1 cells were normal. IL-2-regulated TRG were not affected	MOI was delayed for 1–3 wk but fully developed later with skin and lung inflammation	5–8 wk
Sf.*Ltb4r1^−/−^* [18]	Expanded Th1 and Th2 responses	No effect on MOI Inflammation in skin, lung and liver was similar to Sf mice	4–5 wk
Sf*.Alox5^−/−^* [18]	Th1 response remained high and Th2 response was further enhanced	No effect on MOI	3 wk
Sf*.Il10^−/−^* [18]	Th1 and Th2 remained high	No effect on MOI	3–4 wk
Sf*.Cx3cr1^gfp/gfp^* [18]	Th1 and Th2 remained high	No effect on MOI	3–4 wk

* Not described; ** TCR Tg should have prolonged the lifespan.
